# The combination of dynapenia and abdominal obesity as a risk factor for worse trajectories of IADL disability among older adults

**DOI:** 10.1016/j.clnu.2017.09.018

**Published:** 2017-10-02

**Authors:** Tiago da Silva Alexandre, Shaun Scholes, Jair Licio Ferreira Santos, Yeda Aparecida de Oliveira Duarte, Cesar de Oliveira

**Affiliations:** a Department of Epidemiology and Public Health, University College London, London, United Kingdom; b Department of Gerontology, Federal University of São Carlos, São Carlos, Brazil; c Department of Social Medicine, University of São Paulo, Ribeirão Preto, Brazil; d Department of Medical-Surgical Nursing, University of São Paulo, Sao Paulo, Brazil

**Keywords:** Handgrip, Dynapenia, Weakness, Waist circumference, Obesity, Disability

## Abstract

**Background/Objectives:**

The concept of dynapenic obesity has been gaining great attention recently. However, there is little epidemiological evidence demonstrating that dynapenic abdominal obese individuals have worse trajectories of disability than those with dynapenia and abdominal obesity alone. Our aim was to investigate whether dynapenia combined with abdominal obesity can result in worse trajectories of instrumental activities of daily living (IADL) among English and Brazilian older adults over eight and ten years of follow-up, respectively.

**Methods:**

We used longitudinal data from 3374 participants from the English Longitudinal Study of Ageing (ELSA) and 1040 participants from the Brazilian Health, Well-being and Aging Study (SABE) who were free from disability as assessed by IADL at baseline. IADL disability was defined herein as a difficulty to perform the following: preparing meals, managing money, using transportation, shopping, using the telephone, house cleaning, washing clothes, and taking medications according to the Lawton IADL modified scale. The study population in each country was categorized into non-dynapenic/non-abdominal obese (reference group), abdominal obese, dynapenic and dynapenic abdominal obese according to their handgrip strength (<26 kg for men and <16 kg for women) and waist circumference (>102 cm for men and >88 cm for women). We used generalized linear mixed models with IADL as the outcome.

**Results:**

The estimated change over time in IADL disability was significantly higher for participants with dynapenic abdominal obesity compared to those with neither condition in both cohorts (ELSA: 0.023, 95% CI = 0.012–0.034, p < 0.001; SABE: +0.065, 95% CI = 0.038–0.091, p < 0.001). Abdominal+obesity was also associated with changes over time in IADL disability (ELSA: +0.009, 95% CI = 0.002–0.015, p < 0.05; SABE: +0.021, 95% CI = 0.002–0.041, p < 0.05), which was not observed for dynapenia.

**Conclusions:**

Abdominal obesity is an important risk factor for IADL decline but participants with dynapenic abdominal obesity had the highest rates of IADL decline over time among English and Brazilian older adults.

## 1. Introduction

Muscle strength and muscle mass decline with aging, but heterogeneously in different groups [1]. The complete mechanism that explains this decline and how it affects the muscle function is not fully understood [2,3]. In contrast, there is strong evidence linking low muscle strength with incident mobility limitation, disability in instrumental and basic activities of daily living, and mortality [4–11].

This age-related decline in muscle strength and muscle mass has been accompanied by increasing obesity in older adults globally [12]. The increase in adiposity as a risk factor to low muscle strength and disability and the relationship between adipose tissue and muscle function has attracted interest in recent years [13–19]. The body fat distribution changes with aging resulting in an increase in central adiposity and fat deposition in muscle while there is a reduction in subcutaneous fat [20, 21]. Recent evidence has shown that fatty infiltration of muscle is an important component of low muscle strength and that abdominal obesity can reduce muscle strength through inflammatory and endocrine mechanisms [18–21].

Few studies have investigated the combined associations of abdominal obesity and dynapenia on incident disability [22–25]. The fact that previous studies have analyzed obesity and dynapenia as independent conditions, without taking into account that they can occur simultaneously in older adults, ignores the possibility that strength decline may be associated with obesity. Furthermore, this approach may lead to an overestimation of the association between dynapenia and abdominal obesity with disability.

Therefore, the main aim of this study was to analyze whether dynapenia combined with abdominal obesity was associated with worse trajectories of instrumental activities of daily living (IADL) disability among English and Brazilian older adults who were free from disability, as measured by instrumental activities of daily living, at baseline over a period of eight- and ten-years of follow-up respectively.

## 2. Methods

Data were extracted from the English Longitudinal Study of Ageing (ELSA) and from the Brazilian Health, Well-being and Aging Study (SABE). ELSA is a panel study that began in 2002 with a representative sample of older English adults aged 50 years and over. After baseline, follow-up interviews within ELSA occur every two years and health examinations (i.e. a nurse visit), every four years. SABE is a panel study that began in 2000 with a representative sample of older adults living in Sao Paulo, Brazil, aged 60 years and over. Further information on study design and sampling of both cohorts can be found elsewhere [26,27].

Participants aged 60 years and older in 2004 for ELSA, when anthropometric data were collected for the first time, and in 2000 for SABE were included in this analysis. In ELSA, of 6180 participants interviewed in 2004 with information on instrumental activities of daily living (IADL), 1657 were excluded because they reported at least one disability in IADL at baseline, 1116 were excluded due to missing data for handgrip strength, waist circumference or other covariates, and 33 were excluded due to being underweight (BMI < 18.5 kg/m^2^), resulting in a final analytical sample of 3374 individuals. In SABE, of 2142 participants interviewed in 2000 with IADL information, 863 were excluded because they reported at least one disability in IADL at baseline, 212 were excluded due to missing data for the reasons described above, and 27 were excluded due to being underweight, resulting in a final analytical sample of 1040 individuals. In both of our studies, the handgrip strength and waist circumference measurements were not undertaken for participants who were unable to be in a standing position or were incapable of performing the handgrip test. Underweight older adults were excluded to avoid bias in our results since underweight is an important risk factor for IADL limitation [28].

In ELSA, selected participants were reassessed in 4 and 8 years of follow-up while in SABE they were reassessed in 5 and 10 years of follow-up.

### 2.1. Ethics approval and informed consent

Written informed consent was obtained from all ELSA and SABE participants. The National Research Ethics Service (London Multicenter Research Ethics Committee (MREC/01/2/91)) approved the English Longitudinal Study of Ageing. The Brazilian Human Research Ethics Committee approved Health, Well-being and Aging Study (MS/315/99).

### 2.2. IADL assessment

IADL disability was measured at baseline and at each follow-up visit, in both studies.

Disability was defined herein as a self-reported difficulty to perform the following: preparing meals, managing money, using transportation, shopping, using the telephone, house cleaning, washing clothes, and taking medications according to the Lawton IADL modified scale [29,30]. Eight items were summed to form a scale that ranged from 0 to 8, with 0 representing no disability in IADL. In this analysis, we included only individuals without any IADL disability at baseline.

### 2.3. Anthropometric measurements and classification of the groups

A trained evaluator carried out the waist circumference measurement with a flexible tape placed at the midpoint between the iliac crest and the last rib. Participants remained upright with the arms alongside the body and without the upper portion of their clothes and were instructed to relax the abdomen and the measure was taken at the end of the expiratory phase of a breathing cycle. A waist circumference >88 cm for women and >102 cm for men was considered as abdominal obesity [31].

Grip strength measurements were obtained by a hand held dynamometer (Takei Kiki Kogyio TK 1201 in SABE and Smedley in ELSA). Maximum strength tests were performed with a 1-min rest between tests and the highest value was used. Dynapenia was defined based on two cutoff points for grip strength: <16 kg for women and <26 kg for women [32].

We constructed a four-category time varying variable, based on participants’ dynapenia and abdominal obesity status at each visit. The categories were as follows: non dynapenic/non abdominal obese; abdominal obese only; dynapenic only; and dynapenic abdominal obese. Thus, an individual could be abdominal obese but non dynapenic at one visit and acquire dynapenia at the next. In such circumstances, he/she would be “abdominal obese alone” and “dynapenic abdominal obese” respectively.

### 2.4. Covariates

Covariates included in our analyses were sociodemographic, behavioral characteristics and clinical conditions.

Sociodemographic covariates were age, sex, marital status, income and level of education. Age was categorized into three 10-year age groups. Participants aged 80 years and older were combined into a single age group. Marital status was defined as married (married participants or those in a stable relationship) and not married (separated, divorced or widowed). Socioeconomic status and educational level were measured distinctly in ELSA and SABE. In ELSA, we used household wealth quintiles. Total non-pension household wealth included financial wealth (investments and savings), the value of any business assets, the value of any home and other property (less mortgage), and physical wealth such as jewelry and artwork, net of debt. The three-way educational classification was used to analyze ELSA data: a level lower than “O-level” or equivalent (0–11 schooling years), a level lower than “A-level” or equivalent (12–13 schooling years), and a higher qualification (>13 schooling years). For SABE participants, the Brazilian monthly minimum wage in 2000 (R$151.00 = US$ 84.7) was used to calculate income which was further categorized into three bands: up to two times the minimum wage (−US$ 169.4), two to five (US$ 169.41–423.5) and more than five times the minimum wage (US$ > 423.5). Educational level (in years) was analyzed as a continuous variable.

Smoking status was assessed by asking participants whether they were a current smoker, former smoker or non-smoker. Alcohol consumption was classified as drank never or rarely (even once a week), frequently (2–6 times a week) or daily in both studies. In ELSA, three self-reported questions on the frequency (more than once per week, once per week, one to three times per month, or hardly ever) of participation in vigorous, moderate, and mild physical activities (PA) were used, and two PA groups were created: sedentary lifestyle (no physical activity on a weekly basis), and active (mild, moderate or vigorous physical activity at least once a week). In SABE, sedentary lifestyle was defined as a physical activity level less than three times a week in the last year.

Systemic arterial hypertension, cancer, diabetes, heart disease, lung disease, stroke, osteoarthritis, falls and hospitalization in the previous 12 months were recorded based on self-reports. Hospitalization was not measured in ELSA. Depressive symptoms were defined by the Center for Epidemiologic Studies Depression Scale (CESD) score 4 in ELSA and by the 15-item Geriatric Depression Scale (GDS) score 5 in SABE. Due to the low level of education among the SABE participants, cognitionwas assessed by the modified version of the Mini Mental State Exam (MMSE). This measure has 13 items with a possible total score of 19 points that do not depend upon schooling. Cognitive impairment was defined as a score of ≤ 12 points [33]. In ELSA cognition function was assessed by verbal memory, i.e. immediate and delayed recall. It was assessed by presenting to participants a list of 10 nouns aurally on a computer, one every 2 s. As many words as possible recalled immediately, and after a short delay during which participants carried out the other cognitive tests, were recorded. An overall memory score ranging from 0 to 20 using both the immediate- and delayed-recall results was computed. Body mass index (BMI) was obtained by dividing weight in kilograms by height in meters squared (kg/m^2^) and used as continuous variable. Self-reported near and far vision (good/regular/poor) and hearing (good/regular/poor) were also included in our analyses.

All the covariates included in our analyses represent a broad spectrum of risk factors associated with the progression of IADL disability [30]. All variables were treated in our analyses as time-varying covariates, with the exception of age, sex and level of education.

### 2.5. Statistical analyses

Means, standard deviations (continuous variables) and percentages (categorical variables) were reported for descriptive data at baseline. Differences in baseline characteristics between: (1) included and excluded individuals (due to missing data on hand-grip strength, waist circumference or other covariates), and (2) the four analytical groups classified on the basis of participants’ dynapenia and abdominal obesity status, were assessed using chi square tests, analysis of variance and post hoc Tukey tests. A p value < 0.05 was used to indicate statistical significance.

Cognitive function, income and level of education were considered in this analysis as important risk factors for examining changes in IADL disability over time. As information on these variables was not harmonized, the trajectories of IADL disability were analyzed separately in ELSA and SABE cohorts in order to maintain these important covariates in our models.

To estimate the trajectories in IADL disability we used general linear mixed models using the XTMIXED procedure in Stata 14^®^ SE program (StataCorp, College Station, TX). These models were chosen because they best handle unbalanced data from studies with repeated measures, and they enable the statistical modeling of changes in a time-dependent outcome variable (IADL score), as well as allowing time-dependent change in the magnitude of association between variables [34,35].

Since all participants were free from IADL disability at the baseline visit, the estimates from the mixed models represent the estimated change in IADL score over a follow-up period of one year (i.e. a one-unit increase in time).

We entered a time by dynapenia/abdominal obesity status interaction term into our models to estimate the difference in the change in IADL score for a one-unit increase in time between the dynapenic abdominal obese group and the reference group (neither dynapenia nor abdominal obesity). Similar comparisons to the reference group were made for the dynapenia only and abdominal obesity only groups. The interactions terms therefore enable the pace of change in the IADL score over the follow-up period to vary across the four dynapenia/abdominal obesity groups.

We fitted a series of five sequential models. The unadjusted model (Model 1) contained just the time by dynapenia/abdominal obesity status interaction term. Model 2 was additionally adjusted for socioeconomic covariates. Model 3 was additionally adjusted for behavioral characteristics, Model 4 was additionally adjusted for clinical conditions, and Model 5 was additionally adjusted for BMI since both BMI and waist circumference have been associated with disability [36,37]. The time interactions represent the difference in the annual rate of change (slope) between particular dynapenic abdominal obese group and the reference (neither dynapenia nor abdominal obesity) in disability.

In order to establish whether the association of dynapenia and abdominal obesity only on the trajectories of IADL disability could be overestimated a sensitivity analysis was performed by entering these two variables separately in the models, i.e. dynapenia (no/ yes) and abdominal obesity (no/yes).

## 3. Results

Of the 3374 participants at baseline with no IADL disability and complete data on all covariates in ELSA, 2619 and 2243 were reassessed at 4 and 8 years of follow-up, respectively. Of the 1040 participants at baseline in SABE, 657 and 488 were reassessed at 5 and 10 years of follow-up. Baseline sociodemographic, behavioral and clinical characteristics of both cohorts according to the dynapenia and abdominal obesity groups are shown in Tables 1 and 2.

In our analyses which compared included and excluded individuals by missing data in handgrip strength, waist circumference and other covariates (but all IADL disability free at baseline) the excluded participants in both studies were found to have lower BMI and worse cognitive function. Furthermore, in ELSA, the excluded participants were older, had lower handgrip strength, lower level of education and wealth, living without a partner, smoked more, drank less alcohol, reported more sedentary lifestyle, falls, stroke, were more depressed and had worse vision while, in SABE, they had lower waist circumference (p < 0.05) (Supplemental Table 1).

The prevalence of dynapenic abdominal obesity at baseline was 3.6% (95% CI 3.0–4.3) in ELSA and 6.9% (95% CI 5.4–8.6) in SABE. 3.7% (95% CI 3.1–4.4) of individuals in ELSA were dynapenic compared with 9.4% (95% CI 7.6–11.3) in SABE. In ELSA 46.3% (95% CI 44.6–48.0) were abdominal obese while 41.4% (95% CI 38.4–44.5) had this condition in SABE. The prevalence of non-dynapenic/non-abdominal obese was 46.4% (95% CI 44.7–48.1) in ELSA and 42.3% (95% CI 39.3–45.4) in SABE.

Table 3 shows the fully adjusted general linear mixed models estimated parameters for the change over time in IADL scores as a function of dynapenia and abdominal obesity status over the 8-year period in ELSA and the 10-year period in SABE. In the ELSA cohort, the estimated change over time in IADL score was stable for the reference group (when all covariates were zero or at average values). In other words, according to the estimated coefficients, there was no significant decline in IADL score over time for the following individuals: aged 60–69 years old, males, with higher schooling and those who remained non-dynapenic/non-abdominal obese, married, household wealth = 5th quintile, non-smokers, non-drinkers, non-sedentary lifestyle, no hypertension, no diabetes, no cancer, no lung and heart disease, no stroke, no osteoarthritis, no falls, good perception of vision and hearing, with CESD score <4 points, Mean Memory Score = 20, and BMI = 18.5 kg/m^2^ over time.

Dynapenic abdominal obese participants had a significantly higher rate of increase in IADL disability trajectory over 8-years compared to those who were non-dynapenic non-abdominal obese. The parameter estimate for the difference in slopes was +0.023 IADL points per year (95% CI 0.012–0.034) after adjusting for socioeconomic and behavioral characteristics, clinical conditions and BMI (Table 3, Model 5).

For SABE, the estimated change over time in IADL score was also stable for participants in the reference group. In other words, according to the estimated coefficients there was no significant decline in IADL score over time for the following individuals: aged 60–69 years old, males, with schooling = 16 years and those who remained non-dynapenic/non-abdominal obese, married, income ≥5 Brazilian monthly minimum salary, non-smokers, non-drinkers, non-sedentary lifestyle, no hypertension, no diabetes, no cancer, no lung and heart disease, no stroke, no osteoarthritis, no falls, no hospitalization, good perception of vision and hearing, with GDS score ≥5 points, MMSE ≥13 points, and BMI = 18.5 kg/m^2^ over time.

Dynapenic abdominal obese participants had significantly higher rate of increase in IADL disability trajectory over 10-years than the participants who were non-dynapenic non-abdominal obese. The parameter estimate for the difference in slopes was +0.065 IADL points per year (95% CI 0.038–0.091) after adjusting for socioeconomic and behavioral characteristics, clinical conditions and BMI (Table 3, Model 5).

Our sensitivity analyses showed that by not combining dynapenia and abdominal obesity, and not adjusting for BMI, could result in an overestimation of the parameters for abdominal obesity and dynapenia only on the trajectory of IADL disability in both studies. The models in our sensitivity analysis which adjusted for BMI also showed an overestimation of the parameters on the trajectories of IADL disability for abdominal obesity, but the parameter for dynapenia became statistically insignificant in both studies. This reinforces the importance of the analytical approach adopted in our study: i.e. to consider the combined associations of abdominal obesity and dynapenia on incident disability whilst adjusting for BMI (Table 4).

**Fig. 1:**
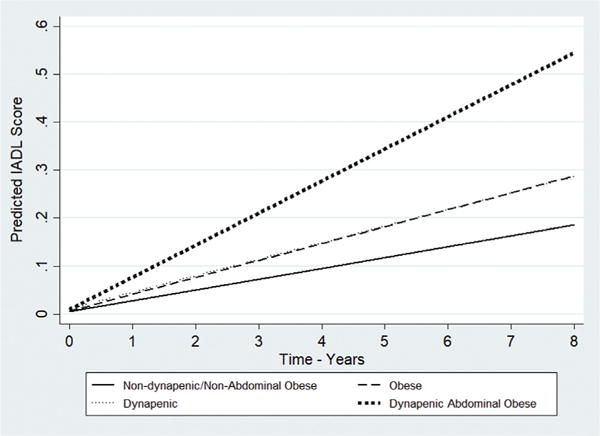
Trajectories of IADL disability according to dynapenia and abdominal obesity status –ELSA study 2004–2012. Predictions for age 60–69, male, married, household wealth = 5th quintile, schooling = higher than A level, drank never or rarely, never smoked, no sedentary lifestyle, no hypertension, no diabetes, no cancer, no lung disease, no heart disease, no stroke, no osteoarthritis, no falls, good perception of vision, good perception of hearing, CESD <4 points, Mean Memory Score = 20 and body mass index = 18.5 kg/m^2^.

**Fig. 2:**
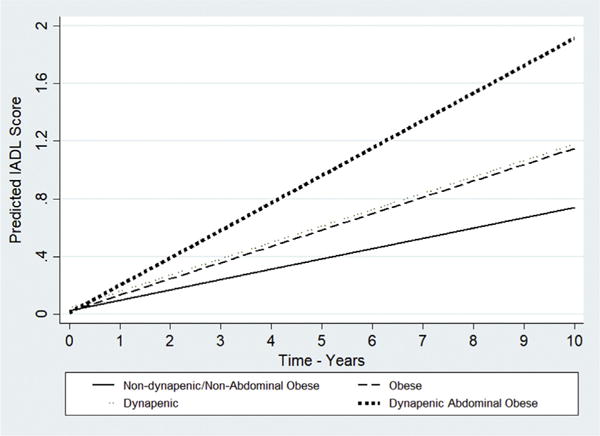
Trajectories of IADL disability according to dynapenia and abdominal obesity status – SABE study 2000–2010. Predictions for age 60–69, male, married, income > US$ 423.5, schooling = 16 years, drank never or rarely, never smoked, no sedentary lifestyle, no hypertension, no diabetes, no cancer, no lung disease, no heart disease, no stroke, no osteoarthritis, no falls, no hospitalization in previous 12 months, good perception of vision, good perception of hearing, GDS <5 points, MMSE ≥ 13 points and body mass index = 18.5 kg/m^2^.

Summarizing, in both the ELSA and SABE cohorts, the estimated IADL disability trajectory in those participants with dynapenic abdominal obesity increased more rapidly over time compared to those with neither condition (Figs. 1 and 2). Although slightly overlapping (as shown in Figs. 1 and 2), compared to those participants with neither condition, participants in both the ELSA and SABE cohorts with abdominal obesity only (but not with dynapenia only) had worse trajectories of IADL disability.

## 4. Discussion

Our main findings showed that dynapenic abdominal obese older adults had worse trajectories of IADL disability over time than non-dynapenic non-abdominal obese individuals. Abdominal obesity only was associated with these trajectories in both English and Brazilian older adults, which was not observed for dynapenia only.

Previous research has found associations between dynapenic abdominal obesity and poorer physical function or disability but none, however, analyzed trajectories. For example, Bouchard et al. analyzing 2039 American men and women aged 55 years and over found in a cross-sectional analysis that dynapenic abdominal obesity was associated with poorer physical function than obesity only and dynapenia only [22]. Rossi et al. using data from 93 to 169 Italian women and men respectively aged between 66 and 78 years from 11 general practitioners found that the risk of worsening disability was higher among dynapenic abdominal obese individuals compared to those who were non-dynapenic non-abdominal obese [23]. Stenholm et al. analyzing six years of followup data from 930 individuals aged 65 years and over from Italy found that obesity (BMI ≥ 30 kg/m^2^) combined with low muscle strength increased the rate of decline in walking speed. These individuals also developed mobility disability, especially those younger than 80 years [24]. Finally, Batsis et al. analyzing 2025 subjects with knee osteoarthritis aged 60 years and over during four years of follow-up in the US, found that obesity only (BMI ≥ 30 kg/m^2^), dynapenia only, and dynapenic obesity was associated in both genders with reduced gait speed at baseline, with the dynapenic obese group presenting the worse performance. At baseline and over the study period, dynapenic obese men had worse performance in a 400-m walking test [25].

The relationship between muscle strength and adiposity is dependent on the measure of obesity used i.e. BMI or waist circumference. Recently, a cross-sectional analysis by Keevil et al. using data from 8441 women and men, aged between 48 and 92 years, from the European Prospective Investigation into Cancer-Norfolk, found that a larger body mass index was associated with higher grip strength. However, high waist circumference was associated with weaker grip strength. Furthermore, they showed that for every 10 cm increase in waist circumference, grip strength was 3.56 kg lower in men and 1.00 kg lower in women. The authors suggested that abdominal fat is the most metabolically active tissue providing a potential mechanism for the association between skeletal muscle and adiposity [38].

It is plausible to assume that increases in central obesity could negatively affect muscle strength with the dynapenic-abdominal obesity group presenting worse IADL disability trajectories. This mechanism is not fully explained, but there is some evidence to support it. For example, adipokines have been shown to be associated with energy balance, immune-modulation, fatty acid and glucose metabolism and inflammatory responses. Physiological and molecular studies have demonstrated receptors to adiponectin and leptin on skeletal muscle cells and shown that their activation promotes decreases in fatty acid deposition, increases the insulin sensitivity and fatty acid oxidation of muscle tissue. However, in obese individuals, skeletal muscle appears to develop resistance to adiponectin and leptin and circulating levels of adiponectin additionally decline [39]. These effects increase skeletal muscle insulin resistance and associated with high expression of circulating cytokines as TNF-α, TNF-β and IL-6, promoted by abdominal obesity, increasing muscle catabolic activity [40]. In addition, TNF is also responsible for depressing the anabolic process and reduce the effect on myelination and repair of damaged axons through reduction of the effects mediated by the insulin-like growth factor-1 (IGF-1) [41]. Moreover, intra- and inter-muscular fat infiltration affecting muscular anatomy and impairing its function has been associated to obesity, particularly to abdominal obesity [42–46].

This study presents some evidence to support the notion that choosing not to separate dynapenic abdominal obesity participants from those with dynapenia and abdominal obesity only leads to an overestimation of the association of these two conditions with worse trajectories of IADL disability. These findings highlight that these changes to body composition can happen simultaneously in later life and are important risk factors for incident disability in these populations.

Several strengths and potential limitations of our study need to be acknowledged. The first strength is the use of easy tools to detect abdominal obesity and dynapenia in clinical practice. Second, the study used data from two large samples of community-dwelling older adults from a developed and developing country with a long period of follow-up. Third, we adjusted our mixed models for a large group of confounding variables associated with IADL disability. Finally, the trajectory analyses were conducted on individuals who were IADL disability free at baseline, allowing us to estimate the associations of these risk factors on IADL disability over time. The identification of potential risk factors could lead to the development of public health strategies to prevent, delay and treat IADL disability.

We would like to acknowledge some limitations. The drop-out rate due to follow-up could be a source of bias. However, this type of bias is unavoidable in longitudinal studies of aging that only include community-dwelling older adults. A further limitation relates to the generalizability of our findings. For example, the disability trajectories of those individuals experiencing cognitive decline in our analyses may be underestimated due to the fact that those participants excluded from our analysis had worse cognitive function than those included in our analytical samples. Similarly, the exclusion of participants who were underweight at baseline may have led to an overestimation of the trajectories for obesity. However, despite the differences between the included and excluded participants in both cohorts with respect to a number of covariates, we were still able to observe significantly worse disability trajectories for dynapenic obese individuals and for those with abdominal obesity only among Brazilian and English older adults. Finally, the lack of information, in both cohorts, about diet, history of obesity, age of onset of obesity and number of years being overweight is also a limitation.

## 5. Conclusions

Abdominal obesity is an important risk factor for IADL decline but participants with dynapenic abdominal obesity had the highest rate of IADL decline over time among English and Brazilian older adults. Our findings highlight the clinical importance of including abdominal obesity and dynapenia in the assessment of disability risk among older adults, particularly when both conditions are present in the same patient and independently of BMI. Therefore, since abdominal obesity and dynapenia are potentially modifiable risk factors, our findings indicate potential paths for preventing or at least delaying the IADL disability process in older adults.

## Supplementary Material

1

## Figures and Tables

**Table 1:** Baseline sociodemographic and behavioral characteristics of 3374 older adults from the ELSA Study (2002) and 1040 elderly from SABE Study (2000) according abdominal obesity and dynapenia status.

	Non-Dynapenic Non-Abdominal Obese	Abdominal Obese	Dynapenic	Dynapenic Abdominal Obese
	ELSA n = 1564 SABE n = 440	ELSA n = 1563 SABE n = 431	ELSA n = 125 SABE n = 97	ELSA n = 122 SABE n = 72
**Sociodemographic variables**				
Age, years				
ELSA	70.9 ± 6.9	71.2 ± 6.5	78.7 ± 8.5^a^^,^^b^	76.5 ± 9.0^a^^,^^b^^,^^c^
SABE	70.0 ± 7.0	69.3 ± 7.0	75.5 ± 7.5^a^^,^^b^	73.8 ± 7.5^a^^,^^b^^,^^c^
Sex (female), (%)				
ELSA	47.8	56.2^a^	52.0	63.1^a^
SABE	36.6	70.1^a^	23.7^a^^,^^b^	80.6^a^^,^^c^
Marital status (married), (%)				
ELSA	71.2	70.6	55.2^a^^,^^b^	54.9^a^^,^^b^
SABE	68.2	53.6^a^	63.9	38.9^a^^,^^b^^,^^c^
Income SABE, (%)				
US$ ≤ 169.4	28.6	30.9	37.1	54.2^a^^,^^b^^,^^c^
>US$ 169.4 and US$ ≤ 423.5	30.7	26.7	34.0	20.8^a^^,^^b^^,^^c^
>US$ 423.5	27.5	23.9	19.6	8.3^a^^,^^b^^,^^c^
Unreported	13.2	18.5	9.3	16.7^a^^,^^b^^,^^c^
Household wealth ELSA, (%)				
5st quintile (highest quintile)	9.5	14.3^a^	24.0^a^^,^^b^	15.6^a^^,^^b^
4nd quintile	15.0	18.2^a^	22.4^a^^,^^b^	27.1^a^^,^^b^
3th quintile	20.3	22.6^a^	15.2^a^^,^^b^	22.1^a^^,^^b^
2th quintile	23.3	22.0^a^	23.2^a^^,^^b^	26.2^a^^,^^b^
1th quintile (lowest quintile)	30.7	21.9^a^	14.4^a^^,^^b^	8.2^a^^,^^b^
Unreported	1.2	1.0^a^	0.8^a^^,^^b^	0.8^a^^,^^b^
Schooling ELSA, (%)				
Higher than A level	29.2	22.2^a^	18.4^a^	9.0^a^^,^^b^
0 level or equivalent	25.5	22.1^a^	16.0^a^	19.7^a^^,^^b^
Less than 0 level or equivalent	45.3	55.7^a^	65.6^a^	71.3^a^^,^^b^
Mean Schooling SABE, years	4.8 ± 4.1	4.3 ± 3.7	3.5 ± 3.4^a^	2.5 ± 2.3^a^^,^^b^
**Behavioral variables**				
Smoking, (%)				
*Never smoked*				
ELSA	41.0	35.7^a^	31.2	36.1
SABE	42.1	60.6^a^	48.4	75.0^a^^,^^c^
*Ex-smoker*				
ELSA	48.1	53.4^a^	57.6	54.9
SABE	37.0	29.5^a^	36.1	20.8^a^^,^^c^
*Current smoker*				
ELSA	10.9	10.9^a^	11.2	9.0
SABE	20.9	9.9^a^	15.5	4.2^a^^,^^c^
Alcohol consumption, (%)				
*Drank never or rarely*				
ELSA	32.4	41.2^a^	40.8	50.8^a^^,^^b^
SABE	81.1	91.2^a^	74.2^b^	95.8^a^^,^^c^
*Drank frequently*				
ELSA	46.5	40.9^a^	43.2	39.3^a^^,^^b^
SABE	9.6	5.1^a^	12.4^b^	1.4^a^^,^^c^
*Drank daily*				
ELSA	21.1	17.9^a^	16.0	9.9^a^^,^^b^
SABE	9.3	3.7^a^	13.4^b^	2.8^a^^,^^c^
Sedentary lifestyle				
ELSA	1.6	1.9	4.0^a^	4.1^a^
SABE	61.8	70.5^a^	77.3^a^	68.1

Data are presented as proportions, means and standard deviation.

a Significantly different from non-dynapenic/non-abdominal obese;

b Significantly different from abdominal obese;

c Significantly different from dynapenic. Statistical significance was set as p < 0.05. NA: Not available.

**Table 2:** Baseline clinical characteristics of 3374 older adults from the ELSA Study (2002) and 1040 elderly from SABE Study (2000) according abdominal obesity and dynapenia status.

	Non-Dynapenic Non-Abdominal Obese	Abdominal Obese	Dynapenic	Dynapenic Abdominal Obese
	ELSA n = 1564 SABE n = 440	ELSA n = 1563 SABE n = 431	ELSA n = 125 SABE n = 97	ELSA n = 122 SABE n = 72
**Clinical conditions**				
Arterial hypertension (yes), (%)				
ELSA	14.8	21.6^a^	16.0	22.1^a^
SABE	39.8	55.5^a^	33.0^b^	65.3^a^^,^^c^
Diabetes (yes), (%)				
ELSA	2.2	4.4^a^	1.6	5.7^a^
SABE	10.5	19.5^a^	13.4	22.2^a^
Cancer (yes), (%)				
ELSA	2.6	4.3^a^	3.2	1.6
SABE	2.7	3.0	1.0	8.3^a^^,^^b^^,^^c^
Lung disease (yes), (%)				
ELSA	9.9	13.0^a^	15.2	14.8
SABE	10.0	7.4	7.2	1.4^a^
Heart disease (yes), (%)				
ELSA	7.2	9.3^a^	12.0	11.5
SABE	12.3	15.1	13.4	20.8^a^
Stroke (yes), (%)				
ELSA	0.8	0.7	0.8	1.6
SABE	3.6	3.3	0.1	5.6^c^
Osteoarthritis, (%)				
ELSA	24.7	34.9^a^	52.0^a^^,^^b^	62.3^a^^,^^b^
SABE	16.6	29.9^a^	18.6^b^	30.6^a^
Falls (yes), (%)				
ELSA	23.9	26.9	29.6	33.6^a^
SABE	22.5	27.8	25.8	31.9
Hospitalization (yes), (%)				
ELSA	NA	NA	NA	NA
SABE	2.7	3.3	3.1	4.2
Mean Memory Score ELSA, points	10.2 ± 3.2	9.9 ± 3.2^a^	8.4 ± 3.7^a^^,^^b^	8.2 ± 3.6^a^^,^^b^
Mini Mental State Exam (≤12 points), (%)				
SABE	2.7	2.1	6.2^b^	6.9^b^
Depression, (%)				
ELSA	6.6	10.2^a^	16.8^a^^,^^b^	14.8^a^
SABE	13.2	12.8	8.3	13.9
Perception of hearing, (%)				
*Good*				
ELSA	80.0	80.0	73.6^a^	78.7^c^
SABE	71.4	76.6	70.1	77.8
*Regular*				
ELSA	16.5	15.7	18.4^a^	20.5^c^
SABE	24.5	19.9	21.7	19.4
*Poor*				
ELSA	3.5	4.3	8.0^a^	0.8^c^
SABE	4.1	3.5	8.2	2.8
Perception of vision (%)				
*Good*				
ELSA	91.1	90.5	82.4^a^^,^^b^	83.6^a^^,^^b^
SABE	13.6	9.5	14.5	11.1
*Regular*				
ELSA	7.5	8.3	15.2^a^^,^^b^	16.3^a^^,^^b^
SABE	40.7	46.9	41.2	51.4
*Poor*				
ELSA	1.4	1.2	2.4^a^^,^^b^	0.1^a^^,^^b^
SABE	45.7	43.6	44.3	37.5
Handgrip strength, kg				
ELSA	32.4 ± 9.9	31.6 ± 9.9^a^	16.1 ± 5.9^a^^,^^b^	15.2 ± 5.3^a^^,^^b^
SABE	30.2 ± 8.0	26.6 ± 8.1^a^	19.3 ± 4.7^a^^,^^b^	14.5 ± 4.3^a^^,^^b^^,^^c^
Waist circumference, cm				
ELSA	87.3 ± 8.7	103.6 ± 9.7^a^	85.6 ± 8.9^a^^,^^b^	103.0 ± 10.1^a^^,^^c^
SABE	87.4 ± 8.6	102.8 ± 9.2^a^	87.4 ± 8.3^a^^,^^b^	100.3 ± 8.4^a^^,^^c^
Body Mass Index, kg/m^2^				
ELSA	24.8 ± 2.5	30.5 ± 3.9^a^	24.2 ± 2.6^a^^,^^b^	30.3 ± 4.1^a^^,^^b^^,^^c^
SABE	23.9 ± 2.6	29.7 ± 4.0^a^	23.2 ± 2.5^a^^,^^b^	28.3 ± 3.3^a^^,^^b^^,^^c^

Data are presented as proportions, means and standard deviation.

a Significantly different from non-dynapenic/non-abdominal obese;

b Significantly different from abdominal obese;

c Significantly different from dynapenic. Statistical significance was set as p < 0.05. NA: Not available.

**Table 3:** General linear mixed models estimates for IADL score as a function of dynapenia and abdominal obesity status over a 8-year period in English older adults (N = 3374) and over a 10-year period in Brazilian older adults (N = 1040).

ELSA	Model 1	Model 2	Model 3	Model 4	Model 5
	Parameter Estimated (Lower to Upper 95% CI)				
	N = 3374	N = 3374	N = 3374	N = 3374	N = 3374
Time, years	0.036 (0.030–0.042)*	0.004 (−0.007–0.016)	0.007 (−0.006–0.020)	0.002 (−0.012–0.016)	0.002 (−0.014–0.017)
Time × ND/NAO	Reference	Reference	Reference	Reference	Reference
Time × AO	0.008 (0.002–0.015)*	0.007 (0.001–0.014)*	0.010 (0.004–0.016)*	0.009 (0.003–0.015)*	0.009 (0.002–0.015)*
Time × D	0.015 (0.004–0.026)*	−0.005 (−0.017–0.007)	0.001 (−0.011–0.013)	−0.002 (−0.014–0.010)	−0.005 (−0.017–0.007)
Time × D/AO	0.037 (0.027–0.048)**	0.025 (0.013–0.037)**	0.030 (0.019–0.042)**	0.028 (0.017–0.039)**	0.023 (0.012–0.034)**

SABE	N = 1040	N = 1040	N = 1040	N = 1040	N = 1040

Time, years	0.124 (0.103–0.144)**	0.091 (0.048–0.135)**	0.085 (0.035–0.134)**	0.054 (0.015–0.092)*	0.037 (−0.039–0.112)
Time × ND/NAO	Reference	Reference	Reference	Reference	Reference
Time × AO	0.015 (−0.007–0.038)	0.011 (−0.012–0.034)	0.018 (−0.004–0.040)	0.019 (0.002–0.037)*	0.021 (0.002–0.041)*
Time × D	0.063 (0.039–0.087)**	0.051 (0.027–0.075)**	0.053 (0.029–0.076)**	0.002 (−0.018–0.021)	−0.005 (−0.024–0.015)
Time × D/AO	−0.092 (0.061 0.123)**	0.077 (0.046–0.108)**	0.060 (0.030–0.090)**	0.065 (0.040–0.090)**	0.065 (0.038–0.091)**

* p < 0.05;

** p < 0.001. There is no term representing differences in the IADL score at baseline as all participants had no IADL disability. The terms represents differences in slope between the group in question and the reference. **Model 1** – Unadjusted model; **Model 2** – Adjusted by socioeconomic characteristics; **Model 3** – Adjusted by socioeconomic and behavioral characteristics; **Model 4** – Adjusted by socioeconomic, behavioral and clinical characteristics; **Model 5** – Adjusted by socioeconomic, behavioral, clinical characteristics and Body Mass Index. ND/NAO: Non dynapenic/non abdominal obese; AO: Abdominal obese only; D: Dynapenic only; D/AO: Dynapenic/Abdominal obese.

**Table 4:** General linear mixed models estimate for IADL score as a function of dynapenia and abdominal obesity status over an 8-year period in English older adults (N = 3374) and over a 10-year period in Brazilian older adults (N = 1040) – Sensitivity Analysis.

Explanatory variable	Model 1	Model 2	Model 3	Model 4	Model 5
	Parameter Estimated (Lower to Upper 95% CI)				
ELSA	N = 3374	N = 3374	N = 3374	N = 3374	N = 3374
Time, years	0.035 (0.029–0.040)**	0.003 (−0.009–0.015)	0.006 (−0.007–0.019)	0.001 (−0.013–0.015)	0.001 (−0.015–0.017)
Time × Abdominal Obesity	0.010 (0.004–0.016)**	0.010 (0.004–0.016)*	0.012 (0.006–0.018)**	0.012 (0.006–0.018)**	0.011 (0.005–0.017)**
Time × Dynapenia	0.023 (0.015–0.030)**	0.008 (−0.001–0.016)	0.012 (0.004–0.021)*	0.010 (0.002–0.018)*	0.006 (−0.002–0.014)

SABE	N = 1040	N = 1040	N = 1040	N = 1040	N = 1040

Time, years	0.122 (0.102–0.143)**	0.090 (0.047–0.134)**	0.085 (0.035–0.135)**	0.052 (0.014–0.091)*	0.032 (−0.044–0.108)
Time × Abdominal Obesity	0.019 (−0.001–0.039)	0.015 (−0.005–0.036)	0.015 (−0.004–0.035)**	0.029 (0.013–0.045)**	0.031 (0.013–0.050)**
Time × Dynapenia	0.068 (0.048–0.089)**	0.056 (0.037–0.076)**	0.049 (0.029–0.068)	0.018 (0.002–0.034)*	0.013 (−0.003–0.029)

* p < 0.05;

** p < 0.001. There is no term representing differences in the IADL score at baseline as all participants had no IADL disability. The terms represent differences in slope between the group in question and the reference. **Model 1** – Unadjusted model; **Model 2** – Adjusted by socioeconomic characteristics; **Model 3** – Adjusted by socioeconomic and behavioral characteristics; **Model 4** – Adjusted by socioeconomic, behavioral and clinical characteristics; **Model 5** – Adjusted by socioeconomic, behavioral, clinical characteristics and Body Mass Index.

## Appendix A. Supplementary data

Supplementary data related to this article can be found at https://doi.org/10.1016/j.clnu.2017.09.018.
