# Supplementary material for: The combination of dynapenia and abdominal obesity as a risk factor for worse trajectories of IADL disability among older adults
Source: Clin Nutr. Author manuscript; Available in PMC 2018 Dec 1. (PMC6013360; doi:10.1016/j.clnu.2017.09.018)
Supplement: 1 [file NIHMS949400-supplement-1.docx]

**Supplemental Table 1.** Comparisons between included and excluded participants in ELSA and SABE at baseline.

|  | | **ELSA** | | | | **SABE** | | | |
| --- | --- | --- | --- | --- | --- | --- | --- | --- | --- |
|  | | **Included** | | **Excluded** | | **Included** | | **Excluded** | |
| **Sociodemographic characteristics** | |  | |  | |  | |  | |
| Age, years | | 71.5 ± 7.1* | | 74.0 ± 8.1* | | 70.5 ± 7.3 | | 71.3 ± 7.5 | |
| Sex (female), (%) | | 52.4 | | 52.6 | | 52.3 | | 47.3 | |
| Marital status (married), (%) | | 69.7* | | 55.5* | | 59.7 | | 56.9 | |
| Income SABE, (%) | |  | |  | |  | |  | |
| US$ ≤ 169.4 | | NA | | NA | | 32.1 | | 31.0 | |
| > US$ 169.4 and US$ ≤ 423.5 | | NA | | NA | | 28.7 | | 25.1 | |
| > US$ 423.5 | | NA | | NA | | 23.9 | | 27.2 | |
| Unreported | | NA | | NA | | 15.3 | | 16.7 | |
| Household wealth ELSA, (%) | |  | |  | |  | |  | |
| 5^th^ quintile (highest quintile) | | 25.3* | | 18.9* | | NA | | NA | |
| 4^nd^ quintile | | 22.8* | | 16.7* | | NA | | NA | |
| 3^th^ quintile | | 21.2* | | 17.9* | | NA | | NA | |
| 2^th^ quintile | | 17.2* | | 21.4* | | NA | | NA | |
| 1^st^ quintile (lowest quintile) | | 13.5* | | 25.1* | | NA | | NA | |
| Schooling ELSA, (%) | |  | |  | |  | |  | |
| Higher than A level | | 24.8* | | 18.5* | | NA | | NA | |
| 0 level or equivalent | | 23.4* | | 15.7* | | NA | | NA | |
| Less than 0 level or equivalent | | 51.8* | | 65.8* | | NA | | NA | |
| Mean Schooling SABE, years | | NA | | NA | | 4.3 ± 3.8 | | 4.7 ± 4.6 | |
| **Behavioral characteristics** | |  | |  | |  | |  | |
| Smoking, (%) | |  | |  | |  | |  | |
| Non-smoker | | 38.0* | | 33.4* | | 52.6 | | 44.3 | |
| Former-smoker | | 51.2* | | 50.3* | | 32.7 | | 38.5 | |
| Current smoker | | 10.8* | | 16.3* | | 14.7 | | 17.2 | |
| Alcohol consumption, (%) | |  | |  | |  | |  | |
| Non-drinkers or drank once a week | | 37.5* | | 41.4* | | 85.7 | | 80.7 | |
| Drank frequently | | 43.5* | | 37.9* | | 7.4 | | 10.5 | |
| Drank daily | | 19.0* | | 20.7* | | 6.9 | | 8.8 | |
| Sedentary lifestyle, (%) | | 1.9* | | 4.2* | | 67.3 | | 65.7 | |
| **Clinical Conditions** | |  | |  | |  | |  | |
| Arterial hypertension (yes), (%) | | 18.3 | | 18.9 | | 47.4 | | 51.7 | |
| Diabetes (yes), (%) | | 3.3 | | 4.5 | | 15.3 | | 15.3 | |
| Cancer (yes), (%) | | 3.4 | | 3.8 | | 3.1 | | 5.1 | |
| Lung disease (yes), (%) | | 11.7 | | 10.8 | | 8.1 | | 10.2 | |
| Heart disease (yes), (%) | | 8.5 | | 9.7 | | 14.1 | | 18.6 | |
| Stroke (yes), (%) | | 0.8* | | 2.3* | | 3.3 | | 4.6 | |
| Osteoarthritis (yes), (%) | | 31.8 | | 32.6 | | 23.3 | | 19.7 | |
| Falls (yes), (%) | | 25.9* | | 29.3* | | 25.7 | | 26.1 | |
| Hospitalization (yes), (%) | | NA | | NA | | 3.1 | | 3.8 | |
| Mean Memory Score ELSA, points | | 9.9 ± 3.2* | | 8.4 ± 3.7* | | NA | | NA | |
| Mini Mental State Exam SABE (≤12 points), (%) | | NA | | NA | | 3.1* | | 10.9* | |
| Depression, (%) | | 8.9* | | 12.8* | | 12.6 | | 12.4 | |
| Perception of hearing, (%) | |  | |  | |  | |  | |
| Good | | 79.7 | | 78.7 | | 73.9 | | 74.9 | |
| Regular | | 16.4 | | 17.0 | | 22.0 | | 23.0 | |
| Poor | | 3.9 | | 4.3 | | 4.1 | | 2.1 | |
| Perception of vision (%) | |  | |  | |  | |  | |
| Good | | 90.2* | | 85.9* | | 11.8 | | 17.1 | |
| Regular | | 8.5* | | 10.4* | | 44.1 | | 40.6 | |
| Poor | | 1.3* | | 3.7* | | 44.1 | | 42.3 | |
| Hand grip strength (kg) | | 30.8 ± 10.5* | | 28.6 ± 10.6* | | 26.6 ± 8.9 | | 25.3 ± 8.2 | |
| Waist circumference (cm) | | 95.4 ± 12.3 | | 95.2 ± 14.2 | | 94.6 ± 11.6* | | 86.2 ± 13.8* | |
| Body Mass Index, kg/m^2^ | | 27.7 ± 4.3* | | 27.1 ± 4.9* | | 26.5 ± 4.4* | | 23.1 ± 14.9* | |

Data are presented as proportions, means and standard deviation. Statistical significance was set as p < 0.05*. NA: Not Applicable.
